# OpenProt 2.0 builds a path to the functional characterization of alternative proteins

**DOI:** 10.1093/nar/gkad1050

**Published:** 2023-11-13

**Authors:** Sébastien Leblanc, Feriel Yala, Nicolas Provencher, Jean-François Lucier, Maxime Levesque, Xavier Lapointe, Jean-Francois Jacques, Isabelle Fournier, Michel Salzet, Aïda Ouangraoua, Michelle S Scott, François-Michel Boisvert, Marie A Brunet, Xavier Roucou

**Affiliations:** Department of Biochemistry and Functional Genomics, Université de Sherbrooke, 3201 Jean Mignault, Sherbrooke, QC J1E 4K8, Canada; Department of Biochemistry and Functional Genomics, Université de Sherbrooke, 3201 Jean Mignault, Sherbrooke, QC J1E 4K8, Canada; Department of Biochemistry and Functional Genomics, Université de Sherbrooke, 3201 Jean Mignault, Sherbrooke, QC J1E 4K8, Canada; Center for Computational Science, Université de Sherbrooke, Sherbrooke, QC J1K 2R1, Canada; Department of Biology, Université de Sherbrooke, Sherbrooke, QC J1K 2R1, Canada; Center for Computational Science, Université de Sherbrooke, Sherbrooke, QC J1K 2R1, Canada; Department of Pediatrics, Medical Genetics Service, Université de Sherbrooke, Sherbrooke, QC J1H 5N4, Canada; Department of Biochemistry and Functional Genomics, Université de Sherbrooke, 3201 Jean Mignault, Sherbrooke, QC J1E 4K8, Canada; INSERM U1192, Laboratoire Protéomique, Réponse Inflammatoire & Spectrométrie de Masse (PRISM), Université de Lille, F-59000 Lille, France; INSERM U1192, Laboratoire Protéomique, Réponse Inflammatoire & Spectrométrie de Masse (PRISM), Université de Lille, F-59000 Lille, France; Informatics Department, Université de Sherbrooke, Sherbrooke, QC J1K 2R1, Canada; Department of Biochemistry and Functional Genomics, Université de Sherbrooke, 3201 Jean Mignault, Sherbrooke, QC J1E 4K8, Canada; Centre de Recherche du Centre Hospitalier Universitaire de Sherbrooke (CRCHUS), Sherbrooke, QC J1H 5N4, Canada; Centre de Recherche du Centre Hospitalier Universitaire de Sherbrooke (CRCHUS), Sherbrooke, QC J1H 5N4, Canada; Department of Immunology and Cellular Biology, Université de Sherbrooke, Sherbrooke, QC J1E 4K8, Canada; Centre de Recherche du Centre Hospitalier Universitaire de Sherbrooke (CRCHUS), Sherbrooke, QC J1H 5N4, Canada; Department of Pediatrics, Medical Genetics Service, Université de Sherbrooke, Sherbrooke, QC J1H 5N4, Canada; Department of Biochemistry and Functional Genomics, Université de Sherbrooke, 3201 Jean Mignault, Sherbrooke, QC J1E 4K8, Canada; Centre de Recherche du Centre Hospitalier Universitaire de Sherbrooke (CRCHUS), Sherbrooke, QC J1H 5N4, Canada

## Abstract

The OpenProt proteogenomic resource (https://www.openprot.org/) provides users with a complete and freely accessible set of non-canonical or alternative open reading frames (AltORFs) within the transcriptome of various species, as well as functional annotations of the corresponding protein sequences not found in standard databases. Enhancements in this update are largely the result of user feedback and include the prediction of structure, subcellular localization, and intrinsic disorder, using cutting-edge algorithms based on machine learning techniques. The mass spectrometry pipeline now integrates a machine learning-based peptide rescoring method to improve peptide identification. We continue to help users explore this cryptic proteome by providing OpenCustomDB, a tool that enables users to build their own customized protein databases, and OpenVar, a genomic annotator including genetic variants within AltORFs and protein sequences. A new interface improves the visualization of all functional annotations, including a spectral viewer and the prediction of multicoding genes. All data on OpenProt are freely available and downloadable. Overall, OpenProt continues to establish itself as an important resource for the exploration and study of new proteins.

## Graphical Abstract

**Graphical Abstract:**
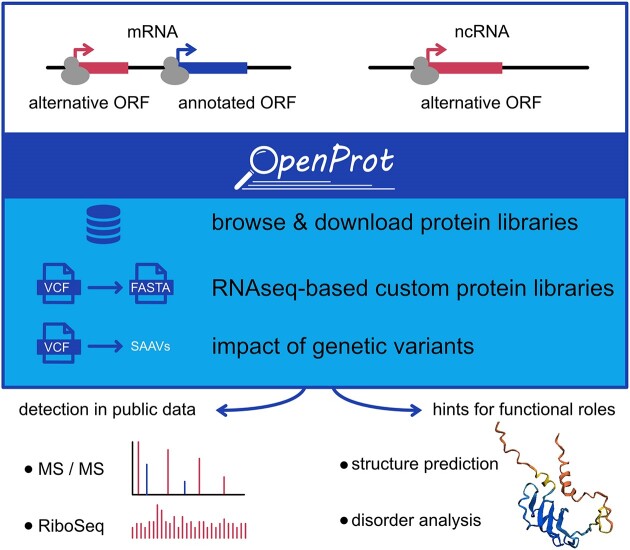

## Introduction

**Figure 1.**
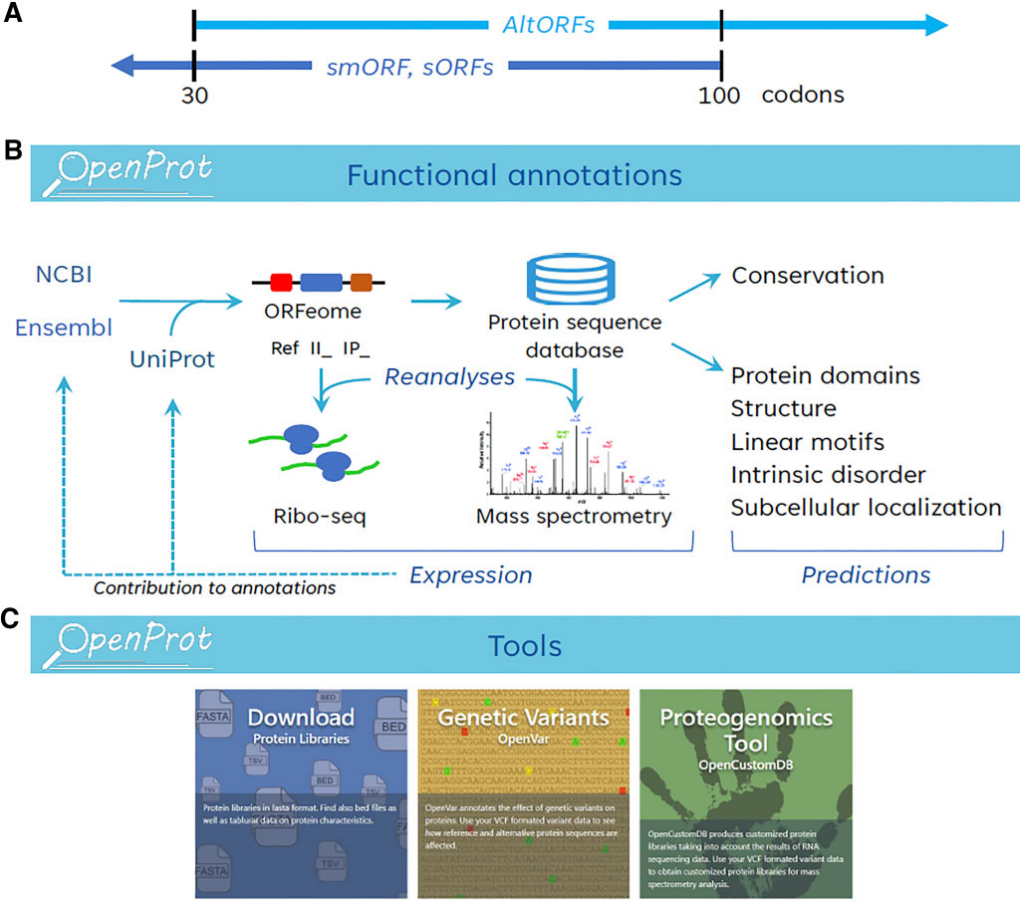
(**A**) AltORFs are defined as ORFs with a minimum size of 30 codons (including the stop codon) currently unannotated in RefSeq and Ensembl, AltORFs can be larger than 100 codons. The longest AltORFs in human has 13 226 codons (II_4421741). Small ORFs (smORFs or sORFs) are typically 100 codons or less in length. (**B**) OpenProt functional annotations. Interrogation of Ensembl and RefSeq transcripts results in the identification of annotated ORFs (or RefORFs) and unannotated ORFs (or AltORFs). The corresponding proteins are classified as RefProts, novel isoforms (accession II_) or AltProt (accession IP_). Reanalyses of ribo-seq and mass spectrometry-based proteomics datasets with the OpenProt databases provide expression evidence and contribute to the addition of AltProts and novel isoforms into standard databases (dotted arrows; Table 1). Functional predictions help identify AltProts and novel isoforms with potential biological activity. (**C**) OpenProt tools. Protein sequence databases are available for download. OpenVar is a genomic variant annotator able to handle multiple ORFs in a single transcript. OpenCustomDB allows users to build customized protein databases from RNAseq data, integrating genetic variants in RefProts, AltProts and novel isoforms.

Mass spectrometry (MS)-based proteomics, ribosome profiling (or ribo-seq), and evolutionary analyses concur toward the existence of proteins translated from noncanonical or alternative open reading frames (ORFs) (1,2). Alternative ORFs (or AltORFs) are ORFs that are not currently annotated in conventional databases, including NCBI RefSeq and Ensembl; they are present within UTRs or overlap the reference ORF in a different reading frame of mRNAs, or within RNAs annotated as non-coding (ncRNA). When present in an mRNA, AltORFs are generally smaller than the annotated reference ORF (RefORF) since RefORFs are typically the longest ORF in the mRNA. AltORFs includes a large fraction of small ORFs or short ORFs (Figure 1A). However, in contrast to small ORFs or short ORFs which are usually limited to ORFs below 100 codons, there is no maximum size restriction for AltORFs. To maintain tractable downstream analyses it is necessary to limit the size of the total AltORFeome, therefore only AltORFs larger than 29 codons are considered. Investigations into this largely unexplored orfeome and proteome require specific resources to provide deeper annotations essential for proteogenomics strategies.

OpenProt annotations (2) stem from challenging long-standing dogmas, including a single protein-coding ORF -typically the longest- in mRNAs, the 100-codon threshold for a functional ORF, the absence of protein-coding ORFs in ncRNAs, and the systematic annotation of pseudogenes-derived RNAs as ncRNAs. A relaxation of these conventional rules is essential to create customized databases for discovering alternative proteins (AltProts) and initiating their functional characterization. From all possible ORFs larger than 29 codons within the transcriptome of RefSeq and Ensembl and without a priori regarding the RNA biotype, OpenProt annotates three types of proteins (Figure 1B): (i) RefProts, also known as reference proteins, comprise well-identified proteins annotated in NCBI RefSeq, Ensembl, and/or UniProt. (ii) Novel isoforms (accession II_) represent unannotated proteins that exhibit a substantial sequence similarity to a RefProt encoded in the same gene. (iii) Finally, AltProts (accession IP_) denote unannotated proteins that lack significant identity with any RefProt associated with the same gene. Using these new annotations, OpenProt reanalyses ribo-seq and MS-based proteomics studies to provide evidence of expression and display protein domains and conservation (Figure 1B).

Since its previous release in 2021 (v1.6) (3), 4587 AltProts and 9163 novel isoforms have changed to RefProts following their annotation in UniProt, NCBI RefSeq and/or Ensembl, illustrating both the impact of the alternative and small ORFs community and the high-standard OpenProt annotations (Table 1). OpenProt users have provided constructive feedback based on their experience using the site, and their comments have been instrumental in several new developments in this update. Here, we present OpenProt v2.0 which incorporates transcript expression, structure predictions, intrinsically disordered regions, and short linear motifs. Also implemented are a new MS data analysis pipeline that includes machine learning-based peptide-spectrum match rescoring to improve identification rates, and a frequently requested mass spectrum viewer to explore MS evidence of each detected protein. OpenProt v2.0 also makes available OpenVar, a genomic variant annotator and effect predictor for multiple ORFs on single transcripts, and OpenCustomDB, a tool to generate RNAseq-derived personalized databases accounting for alternative ORFs and their variants (Figure 1C). All ORFs prediction analyses were recomputed with the March 2023 RefSeq (217) and Ensembl (106) annotations, and all prediction and expression analyses were computed with the latest version of the softwares. Overall, OpenProt now integrates both functional annotations and specific tools to explore AltORFs and AltProts. Finally, the platform features a new web interface to facilitate the exploration of the data.

**Table 1.** OpenProt is a significant source of information for conventional annotations

Species	AltProt v1.6 to RefProt v2.0 (#)	Novel isoform v1.6 to RefProt v2.0 (#)	Total changes (#)
*H. sapiens*	2441	5316	7757
*P. troglodytes*	106	440	546
*M. musculus*	1326	1898	3224
*R. norvegicus*	218	430	648
*B. taurus*	1	18	19
*O. aries*	33	639	672
*D. rerio*	1	47	48
*D. melanogaster*	453	302	755
*C. elegans*	8	73	81
*S cerevisiae*	0	0	0
*All 10 species*	4587	9163	13 750

## Updates and new developments

### Genome annotation, ORFs prediction and genome browser

OpenProt 2.0 is based on genome annotations from January 2023 for each species. Table 2 lists genome assemblies, annotation releases, and the number of predicted ORFs categorized as RefORF, novel isoforms, and AltORFs (Supplementary S1 and S2). Two additional species were added: Xenopus tropicalis and Arabidopsis thaliana. Data for all species can be downloaded as tsv, fasta or bed files. All ORF and protein predicted and annotated by OpenProt are displayed in a new genome browser which includes tracks for transcripts and for all predicted proteins coded by the corresponding transcripts.

**Table 2.** OpenProt annotations v2.0

		Annotations		ORFeome (Ensembl & NCBI RefSeq)			
Species	Genome assembly	Ensembl	NCBI RefSeq	RefProt	AltProt	Novel isoforms	Total
*H. sapiens*	GRCh38.p13	GRCh38.106	GRCh38.p13	247352	595788	70786	913926
*P. troglodytes*	Pan_tro_3.0	Pan_tro_3.0.106	Pan_tro_3.0	140749	258489	16329	415567
*R. norvegicus*	mRatBN7.2	mRatBN7.2.106	mRatBN7.2	89015	328386	16499	433900
*M. Musculus*	GRCm39	GRCm39.106	GRCm39	125814	501899	47930	675643
*B. taurus*	ARS-UCD1.2	ARS-UCD1.2.106	ARS-UCD1.2	76027	206599	11419	294045
*O. aries*	Oar_v3.1	Oar_v3.1.106	Oar_v3.1	97620	284298	14367	396285
*X. tropicalis*	UCB_Xtro_10.0	UCB_Xtro_10.0.107	UCB_Xtro_10.0	72622	217436	8666	298724
*D. rerio*	GRCz11	GRCz11.106	GRCz11	83325	208073	11710	303108
*D. melanogaster*	Release 6 plus ISO1 MT	BDGP6.32.106	BDGP6.32	42682	82406	1909	126997
*C. elegans*	WBcel235	WBcel235.106	WBcel235	29502	66719	2978	99199
*S. cerevisiae*	R64-1-1	R64-1-1.106	R64	4859	10268	1909	17036
*A. thaliana*	TAIR10	TAIR10.54	TAIR10	114859	119499	5031	239389

### Additional functional annotations

To determine which predicted proteins annotated by OpenProt are likely functional components of cellular processes it is useful to curate an assortment of functional analyses on these sequences (Supplementary S1). Results from the analyses described below are made available on the OpenProt website for browsing and download.

### Tissue-specific transcript expression

Since OpenProt's functional annotations are primarily transcriptome-based, users have requested the incorporation of transcript expression data. These data were obtained for all transcripts annotated in the Genotype-Tissue Expression (GTEx) portal, a resource reporting the landscape of gene expression in multiple human tissues (4) and are displayed as transcript per million.

### Structure prediction

For a large number of novel isoforms and AltProts annotated in OpenProt, the ability to predict the structure from their amino acid sequence represents an important step in their functional characterization. AlphaFold2 preprocesses multiple sequence alignments and provides accurate predictions for proteins with sequence homologs (5). Among the factors that limit the accuracy of AlphaFold predictions, a minimum of 30 sequences in the multiple sequence alignments is important to generate confident structure predictions. This is a challenge for AltProts which have no significant homology to other proteins. For proteins with multiple sequence alignments with less than 30 sequences, OmegaFold was used (6). OmegaFold is a new protein structure prediction method that does not rely on multiple sequence alignments but on amino-acid sequence only. Both methods predict structures with a confidence measure called the predicted local distance difference test or pLDDT. A total of 96 800 human AltProts and 29 017 novel isoforms display a high (90 > pLDDT > 70) to very high (pLDDT > 90) confidence score (Supplementary S3, panel A). This reliable estimate of the degree of agreement between predicted and experimental structure for several thousand proteins suggests that these newly predicted proteins may adopt specific structures and perform specific biological functions. This observation is expected to stimulate further research into the molecular function of these proteins.

### Intrinsically disordered regions

Proteins with intrinsically disordered regions (or IDRs) have important roles in many biological processes and diseases (7). The research community has often raised the possibility that AltProts may be unstructured and enriched in IDRs compared to RefProts. Indeed, a large fraction of AltProts have a pLDDT < 50 which could indicate a propensity to display IDRs (Supplementary S3, panel A). We used the flDPnn computational tool (8) to predict disorder and disorder function in all protein sequences. Typically, functionally relevant IDRs are defined as containing 30 consecutive residues or more (9). Since the shortest proteins in OpenProt are 29 amino acids long, we computed IDRs with a minimum of 29 disordered consecutive residues and found that 15.7% of RefProts, 22.09% of AltProts and 24.05% of novel isoforms contain at least one IDR (Supplementary S3, panel B).

### Short linear motifs

Short linear motifs (SLiMs) are small functional motifs of three to about 20 amino acids with critical biological functions, usually located in IDRs (10). All proteins were analyzed with the Eukaryotic Linear Motif resource (11) and SLiMs with at least one amino acid predicted as ordered were filtered out.

### Subcellular localization prediction

DeepLoc 2.0 was used to predict the subcellular localization from the sequences of all proteins (12).

### Mass spectrometry data analysis

**Figure 2.**
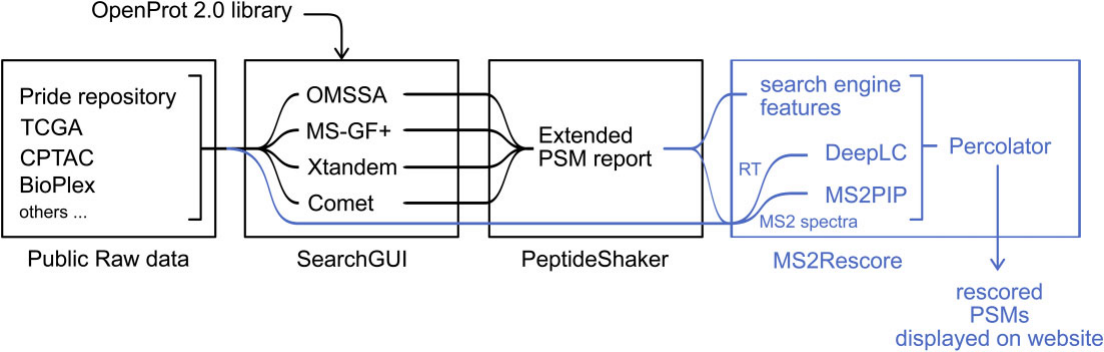
Publicly available MS raw datasets are downloaded from various sources. The OpenProt v2.0 MS pipeline integrates deep learning-based predictions of peptide properties, including retention time (RT) and MS2 spectra intensity predictors within the Percolator postprocessing tool (blue) into the previous MS pipeline (black). PSM, peptide spectrum match; RT, retention time.

**Figure 3.**
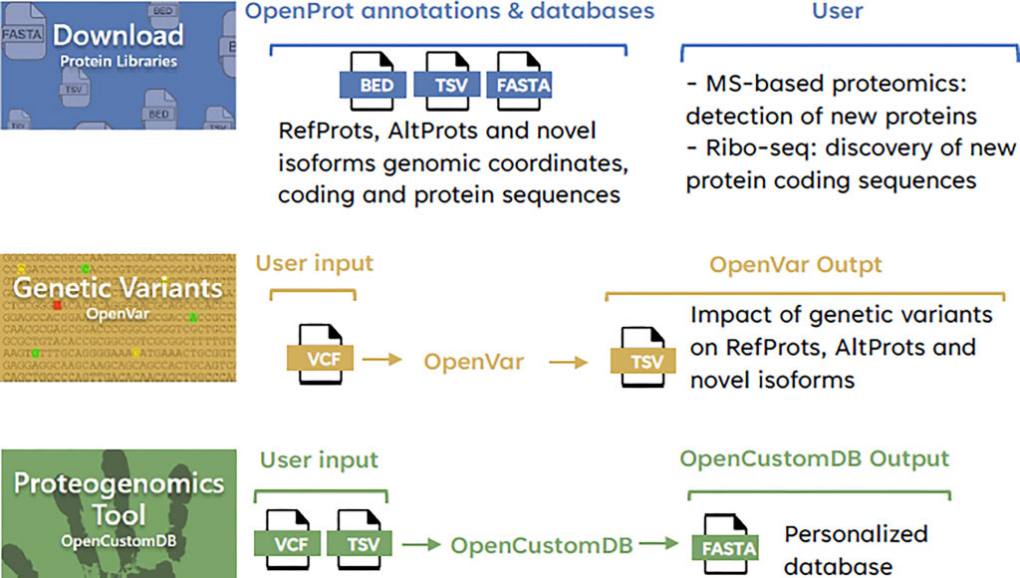
Several platforms are now available on OpenProt v2.0. Annotations for 12 different species are available for download in different formats. Users may upload a variant calling file (VCF) and run the OpenVar genomic annotator. OpenVar provides a listing of impact (i.e. modifier, low, moderate, high) on AltProts and novel isoforms, in addition to RefProts. OpenCustomDB generate sample-specific protein sequence databases from RNAseq data. Users need to upload two files: a VCF and a transcription expression file (processed tab-separated TSV).

An important goal of OpenProt is the discovery of novel proteins, i.e. to gather evidence of expression of predicted proteins both at the translational and protein levels by reanalyzing large-scale Ribo-seq and MS-based proteomics data (Supplementary S4). For typical bottom-up proteomics, the size of protein sequence databases integrating RefProts, novel isoforms, and AltProts is an important challenge, as it introduces greater uncertainty between peptide-spectrum matches, ultimately leading to a decreased number of identified peptides. In this update, we introduced deep learning predictions with MS^2^Rescore (13) to improve the rescoring of peptide spectrum matches and increase peptide identification rates (Figure 2). Raw data extracted from publicly available datasets (Supplementary S4) were analyzed with 4 search engines (X!Tandem, MS-GF+, Comet and OMSSA) with the interface SearchGUI (version 4.2.8). Identifications were aggregated into a single identification set using PeptideShaker (version 2.2.23) as previously described (2). Peptide-spectrum matches were rescored using a combination of MS2PIP, a spectral intensity predictor (14), a high-performance liquid chromatography retention time predictor, DeepLC (15), and the postprocessing tool Percolator (16) within MS2Rescore (13) as previously described (17) (Figure 3). This method improves the sensitivity of peptide spectrum matching and allows the identification of a larger number of peptides. Using the same MS datasets from the previous version of OpenProt 10047 alternative proteins that initially were only identified by one peptide now have two or more peptides detected. Peptide spectrum matches were selected by applying a FDR <0.01%, and peptides unicity from AltProts and novel isoforms was checked against Ensembl, RefSeq and UniProt.

### OpenProt new tools

Two tools accessible both as a Python package and as a user-friendly web-based platform, OpenVar and OpenCustomDB, have been added and are accessible from the Home Page (Figure 3).

OpenVar is a new genomic variant annotator and effect predictor for multiple ORFs on single transcripts, including overlapping ORFs, that extends genomic variant analysis to include non-canonical ORFs (18). OpenVar predicts the effect of genomic variants in all ORFs annotated in OpenProt. Standard variant calling files are used as input.

OpenCustomDB utilizes sample-specific RNA-seq data to detect genetic variants within all ORFs annotated in OpenProt and builds customized protein sequence databases (19). With default parameters, the custom database is configured to accommodate a maximum of 100 000 proteins, with the number of transcripts restricted accordingly. Users can provide a transcript inclusion or exclusion list rather than rely solely on the expression level threshold. Standard variant calling files and transcript expression files are used as input.

### Database development

All data were generated using in-house Python (version 3.6.9) scripts and stored in a SQLite database (version 3.38).

### Web server

**Figure 4.**
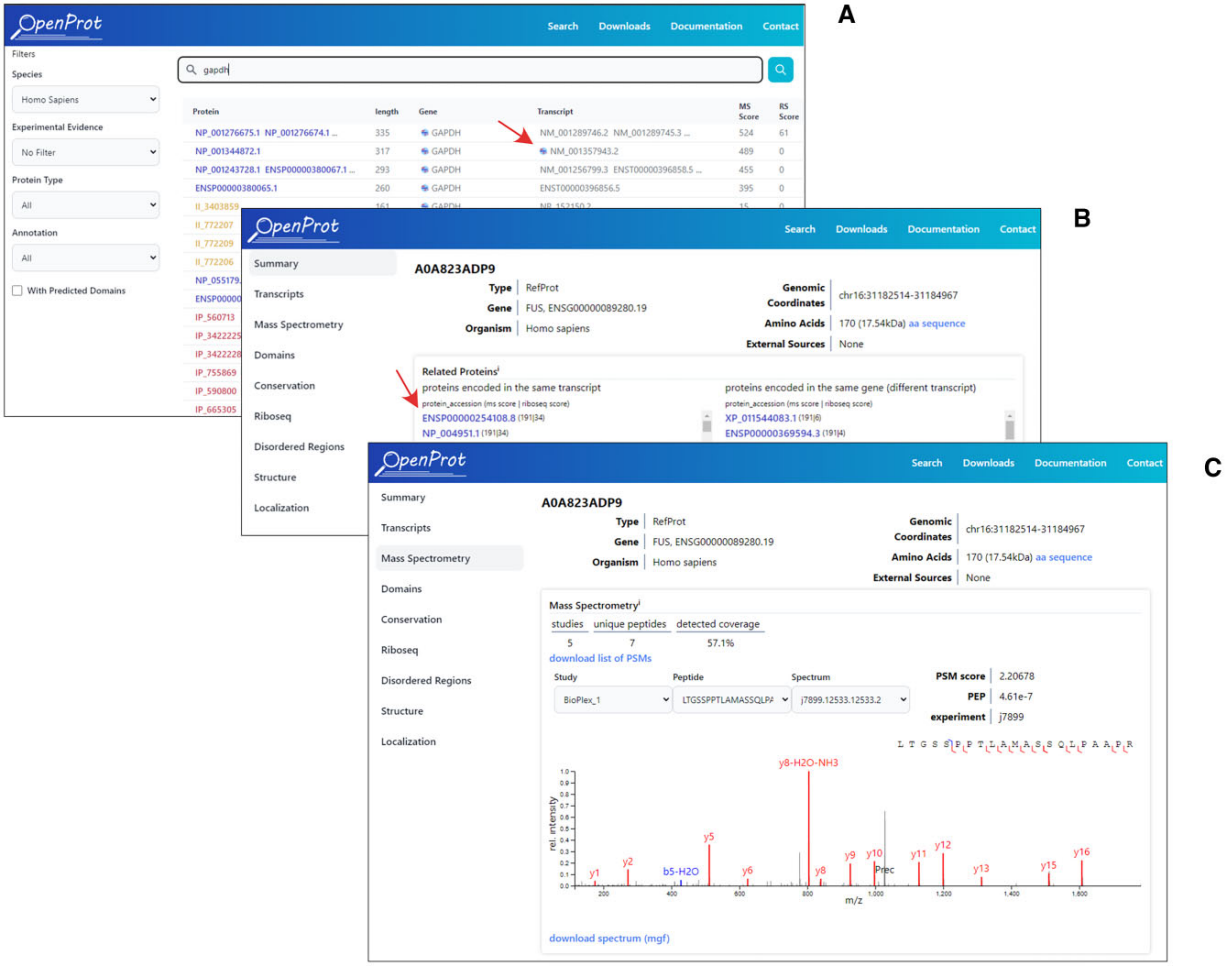
Screenshots of search results and details page. (**A**) Search results for a gene (here *GAPDH*), transcript accession, or protein accession. Each row in the result table always represents a protein regardless of the input. The red arrow points to a multi-coding icon for a specific transcript. (**B**) Details page for protein A0A823ADP9 (accession UniProt), a second protein coded in the *FUS* gene. A list of proteins encoded by the same transcript is shown including the first protein annotated in the *FUS* gene (indicated by the red arrow). (**C**) A spectral viewer is now available for the visualization of MS data. A specific spectrum is shown for protein A0A823ADP9.

The search query can include a gene name, a protein, or a transcript accession from UniProt, RefSeq NCBI, Ensembl, or OpenProt. When searching for a gene name, the search results page displays all proteins associated with this gene, including the proteins already annotated in UniProt, RefSeq, and Ensembl, as well as AltProts and novel isoforms predicted by OpenProt. Proteins are sorted according to experimental evidence and transcripts that encode more than one protein for which a certain level of evidence is available are marked by an icon to indicate their likely multi-coding status (Figure 4A). The level of evidence required for each protein is at least 2 unique peptides detected in the MS dataset or 1 peptide plus one detection in a Ribo-seq dataset. From the search results page a details page displaying the functional annotations for each protein can be accessed. Figure 4B shows the summary tab of the details page for protein A0A823ADP9, previously annotated by OpenProt with the accession ID IP_243680 and now annotated by UniProtKB. Thus, protein A0A823ADP9 is a second reference protein coded by the FUS gene.

### Mass spectrum viewer

Typical MS/MS proteomics analyses produce both spectra of fragmented ions, and their corresponding matched peptides. Previously, OpenProt displayed matched peptides only, but the new version now includes a spectrum viewer that allows the visualization and download of MS2 spectra in the browser (Figure 4C). This direct access to analytical results provides the user with more detailed information and enables a more accurate assessment of the quality of the evidence.

### Links to other repositories

Some repositories, including uORFdb (20), sORFs (21), SmProt (22) and nuORFdb (23) list short ORFs that are predicted or with evidence of ribo-seq. For AltORFs shared between OpenProt and these repositories, the repository-specific accession identifier and link, when available, are provided on each protein details page. OpenProt shares 19 651, 871, 33 552 and 21 337 human AltORFs with uORFdb, sORFs, SmProt and nuRFFdb.

## Conclusion

To the best of our knowledge, no other resource provides functional annotation of RNAs by allowing the presence of multiple ORFs on the same RNA independently of its biotype, thus facilitating functional exploration of proteins encoded in non-canonical coding sequences and recognizing the multicoding (polycistronic) nature of eukaryotic RNAs. Over the last three years, we have carried out significant development of OpenProt. On the functional annotations side, we have added predictions for the structure, subcellular localization, intrinsic disorder, and the presence of short linear motifs. Although most of the additional information provided in this update concerns human annotations, this information will also be available in the short term for the other species available in OpenProt. We have also incorporated deep learning-based predictions of peptide properties in the MS analysis pipeline to improve peptide identifications and added a spectral viewer. Two new tools have been made available to allow users to build their own customized protein sequence databases from RNAseq data incorporating RefProts, AltProts and novel isoforms, including genetic variants. With the increasing complexity of the resource, we also released a new website to facilitate access to the different data and tools.

The prediction of protein-protein complexes using molecular surface interaction fingerprinting (24) is one of the next planned new developments for OpenProt. All the resources available on OpenProt should help users to detect and investigate the function of new proteins, thereby improving knowledge of the molecular function of eukaryotic genes.

## Supplementary Material

gkad1050_Supplemental_File

## Data Availability

OpenProt 2.0 is freely available without registration or login at https://www.openprot.org/.
