# Supplementary material for: OpenProt 2.0 builds a path to the functional characterization of alternative proteins
Source: Nucleic Acids Res. 2023 Nov 13;52(D1):D522–8. doi: 10.1093/nar/gkad1050 (PMC10767855; doi:10.1093/nar/gkad1050)
Supplement: gkad1050_Supplemental_File [file gkad1050_supplemental_file.pdf]

Supplementary Material

*****

TABLE OF CONTENT

Supplementary S1: Summary of the methods.

Supplementary S2: General statistics per species for OpenProt v2.0

Supplementary S3: Protein structure and disorder predictions

Supplementary S4: List of mass spectrometry and ribosome profiling studies incorporated in OpenProt v2.0

*****

Supplementary S1: Summary of the methods

A summary of the methods behind all OpenProt features presented in the initial release was published in the OpenProt 2021 update [1]. We present below some minor modifications in this 2024 update resulting from new annotation releases and the use of the latest version for all software. We also present new developments.

1. **Summary of previous methods with minor modifications**

   a. **OpenProt ORFeome, reference protein, novel isoform, alternative protein -** OpenProt retrieves the transcriptome from both NCBI RefSeq [2] (release 217) and Ensembl [3] (release 106) and predicts ORFs from a 3-frames in silico translation using EMBOSS Transeq (6.6.0) [4]. All ORFs with an ATG initiating codon and a minimal length of 30 codons are annotated and constitutes the OpenProt ORFeome. After in silico translation, three categories of proteins are annotated:

      i. A reference protein (or RefProt) is a protein matching a NCBI RefSeq [2], Ensembl [3] or UniProt [5] (release_2022_06) protein entry. A RefProt is identified with the accession number from NCBI RefSeq, Ensembl and/or UniProt.

      ii. A novel isoform is a protein encoded in the same gene as a RefProt with at least one of the two following features: (a) over 80 % of protein sequence identity with the RefProt over 50% of the length using Basic Local Alignment Search Tool (BLAST); (b) identical genomic coordinates of start or end codon with a sequence identity (EMBOSS Matcher PAM10 matrix score $\leq$ 100) over 20% of the length of the RefProt. A novel isoform is identified with a specific accession number starting with II_.

      iii. An alternative protein (or AltProt) does not have any significant similarity with a RefProt and is identified with a specific accession number starting with IP_.

   b. **Ribosome profiling data reanalysis** - Ribo-seq datasets are downloaded from the Gene Omnibus platform [6] and reanalyzed using the PRICE workflow [7] run with default parameters, except for the FDR set at 1 % (instead of 10%). After filtering out ribosomal RNA reads, PRICE generates a list of ORF candidates based on likely codons. A stringent 1% FDR filter is applied for highly confident translation events.

   c. **Conservation analysis -** Protein sequence homology is assessed using an InParanoid-like workflow [1], distinguishing orthologs (homologous sequences from different species) from paralogs (homologous sequences from the same species but different genes). Orthologs are identified through all-vs-all BLAST searches between species and within the same species for paralogs. Orthology relationships are established with a bit-score above 40 and an overlap exceeding

50% of the queried sequence.

d. **Protein functional domain -** All proteins annotated on OpenProt are scanned for the presence of known functional signatures using InterProScan (5.61-93.0) [8] with the default parameters. Domain predictions as well as gene ontology (GO) and pathway annotations are reported if significant (e-value < 10-3).

2. Updated mass spectrometry data analysis pipeline in the OpenProt

OpenProt re-analyzes mass spectrometry (MS) datasets to gather evidence of expression for every protein annotated in the OpenProt database, *i.e.* RefProts, AltProts and Isoforms. The updated pipeline is described in the main text. It is important to note that when a peptide matches both an AltProt or a novel isoform and a RefProt, it is assigned to the RefProt only.

3. Spectral viewer

A bespoke spectrum viewer was implemented in JavaScript to visualize all spectra that resulted from the mass spectrometry re-analysis. The peak annotations for each spectrum are from the PeptideShaker report of the corresponding analysis.

4. Integration of other predictions

a. Protein structure
MMseqs2 [9] was used to generate large-scale multiple alignment sequences (MSAs). These MSAs serve as inputs for the AlphaFold network, a feature provided by ColabFold with default settings [10]. The installer script was installed within a Singularity image and executed on supercomputers managed by Calcul Québec and the Digital Research Alliance of Canada. For proteins with multiple sequence alignments of less than 30, we utilized OmegaFold the first computational approach that achieved the successful prediction of high-resolution protein structures solely from a single primary sequence [11].

b. Subcellular localization
The DeepLoc 2.0 [12] program was obtained from https://services.healthtech.dtu.dk/cgi-bin/sw_request?software=deeploc&version=2.0&packageversion=2.0&platform=All, installed in an Apptainer container and executed. We used the high-quality (slow) and short output settings. The fasta file generated by OpenProt for all species was given as the input and the csv file of the output is displayed directly on the website.

c. Intrinsic disorder
A Docker image of the flDPnn disorder and disorder function predictor [13]

(https://gitlab.com/sina.ghadermarzi/fldpnn/-/tree/master) was utilized and executed within a Docker container. Input files in Fasta format containing the protein sequences were employed. The resulting output comprises a graph that illustrates the flDPnn score, which is a measure of the probability of disorder (with a threshold of 0.3), along with potential functions such as protein binding, DNA binding, RNA binding, and acting as a linker.

d. Short linear motifs (SLiMs)
We downloaded the tsv file containing all the ELM database Classes using the ELM API (http://elm.eu.org/elms/elms_index.tsv). SLiMs were predicted using the regular expression of the corresponding ELM class. Since this method tends to return a high amount of false positive and true SLiMs are mostly located in disordered regions of a protein [14], we used flDPnn disorder predictions to discard all predicted SLiMs containing at least one residue predicted as ordered.

e. Genotype-Tissue Expression (GTEx)
The data used for the analyses described in this manuscript were obtained from: the GTEx Portal on 07/20/2023 [15]. Expression data from all samples were kept structured at the transcript level rather than aggregated by genes in order to obtain transcript level distributions.

References

[1] Brunet,M.A., Brunelle,M., Lucier,J.F., Delcourt,V., Levesque,M., Grenier,F., Samandi,S., Leblanc,S., Aguilar,J.D., Dufour,P. et al. (2019) OpenProt: a more comprehensive guide to explore eukaryotic coding potential and proteomes. *Nucleic Acids Res.*, **47**, D403–D410.
[2] O'Leary,N.A., Wright,M.W., Brister,J.R., Ciufo,S., Haddad,D., McVeigh,R., Rajput,B., Robbertse,B., Smith-White,B., Ako-Adjei,D. et al. (2016) Reference sequence (RefSeq) database at NCBI: current status, taxonomic expansion, and functional annotation. *Nucleic Acids Res.*, **44**, D733-D745
[3] Cunningham,F., Allen,J.E., Allen,J., Alvarez-Jarreta,J., Amode,M.R., Armean,I.M., Austine-Orimoloye,O., Azov,A.G., Barnes,I., Bennett,R. et al. (2022) Ensembl 2022. *Nucleic Acids Res.*, **50**, D988-D995
[4] Madeira,F., Pearce,M., Tivey,A.R.N., Basutkar,P., Lee,J., Edbali,O., Madhusoodanan,N., Kolesnikov,A. and Lopez,R. (2022) Search and sequence analysis tools services from EMBL-EBI in 2022. *Nucleic Acids Res.*, **50**, W276-W279.
[5] The UniProt consortium (2023) UniProt: the universal protein knowledgebase in 2023. *Nucleic Acids Res.*, **51**, D523-D531.
[6] Barrett,T., Wilhite,S.E., Ledoux,P., Evangelista,C., Kim,I.F., Tomashevsky,M., Marshall,K.A., Phillippy,K.H., Sherman,P.M., Holko,M. et al. (2013) NCBI:GEO: archive for functional genomics data sets-update. *Nucleic Acids Res.*, **41**, D991-D995.
[7] Erhard,F., Halenius,A., Zimmermann,C., L'Hernault,A., Kowalewski,D.J., Weekes,M.P., Stevanovic,S., Zimmer,R., Dölken,L. (2018) Improved Ribo-seq enables identification of cryptic translation events. *Nat. Methods*, **15**, 363–366.
[8] Paysan-Lafosse,T., Blum,M., Chuguransky,S., Grego,T., Pinto,B.L., Salazar,G.A., Bileschi,M.L., Bork,P., Bridge,A., Colwell,L. (2022) InterPro in 2022. *Nucleic Acids Res.*, **51**,

D418-D427.
[9] Steinegger,M. and Söding,J. (2017) MMseqs2 enables sensitive protein sequence searching for the analysis of massive data sets. *Nat. Biotechnol.*, **35**, 1026–1028
[10] Mirdita,M., Schütze,K., Moriwaki,Y., Heo,L., Ovchinnikov,S and Steinegger,M. (2022) ColabFold: making protein folding accessible to all. *Nat Methods*, **19**, 979-682
[11] Wu,R., Ding,F., Wang,R., Shen,R., Zhang,X., Luo,S., Su,C., Wu,Z., Xie,Q., Berger,B. et al. (2022) High-resolution de novo structure prediction from primary sequence. BioRxiv doi: https://doi.org/10.1101/2022.07.21.500999, 22 July 2022, pre-print: not peer-reviewed.
[12] Thumuluri,V., Almagro Armenteros,J.J., Johansen,A.R., Nielsen,H. and Winther,O. (2022) DeepLoc 2.0: multi-label subcellular localization prediction using protein language models. *Nucleic Acids Res.*, **50**, W228–W234.
[13] Hu,G., Katuwawala,A., Wang,K., Wu,Z., Ghadermarzi,S., Gao,J. and Kurgan,L. (2021) flDPnn: Accurate intrinsic disorder prediction with putative propensities of disorder functions. *Nat Comm.*, **12**, 4438.
[14] Kumar,M., Michael,S., Alvarado-Valverde,J., Mészáros,B., Sámano-Sánchez,H., Zeke,A., Dobson,L., Lazar,T., Örd,M., Nagpal,A. et al. (2022) The Eukaryotic Linear Motif resource: 2022 release. *Nucleic Acids Res.*, **50**, D497–D508.
[15] GTEx Consortium (2013) The genotype-tissue expression (GTEx) project. *Nat Genet.*, **45**, 580-585.

Supplementary S2: General statistics per species for OpenProt v2.0

Here are the summary statistics for all species supported in OpenProt v2.0: *Homo sapiens*, *Pan troglodytes*, *Mus musculus*, *Rattus norvegicus*, *Bos taurus*, *Ovis aries*, *Danio rerio*, *Drosophila melanogaster*, *Caenorhabditis elegans* and *Saccharomyces cerevisiae*.

Legend for the pie charts: ncRNA = non-coding RNA; UTR = untranslated region of an mRNA; MS
= mass spectrometry.

*** OpenProt v2.0 general statistics in ***Homo sapiens*** ***

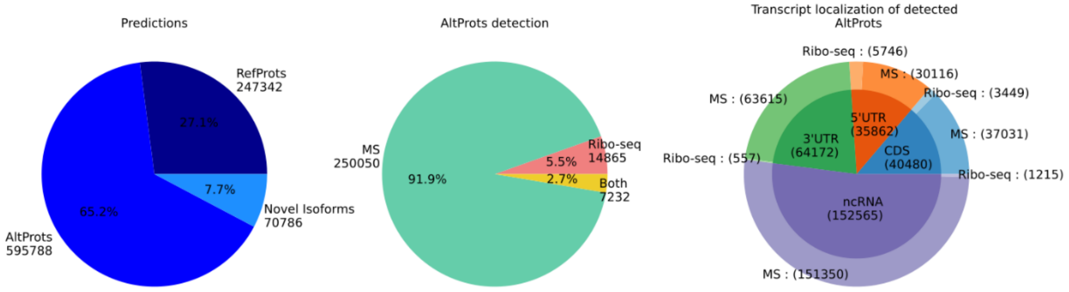

*** OpenProt v2.0 general statistics in ***Pan troglodytes*** ***

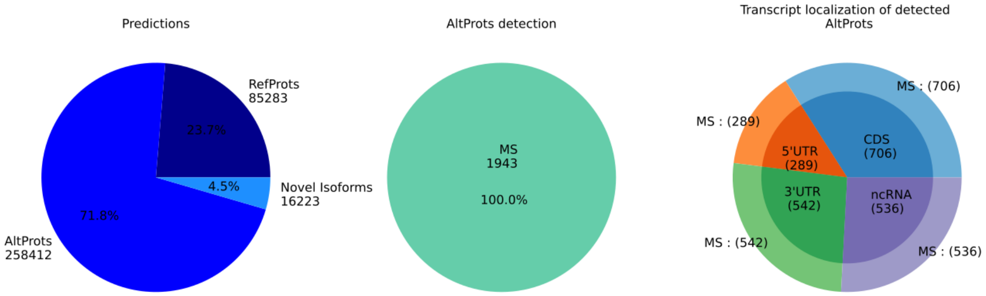

Please note that no ribosome profiling data was available for *Pan troglodytes*.

*** OpenProt v2.0 general statistics in ***Mus musculus*** ***

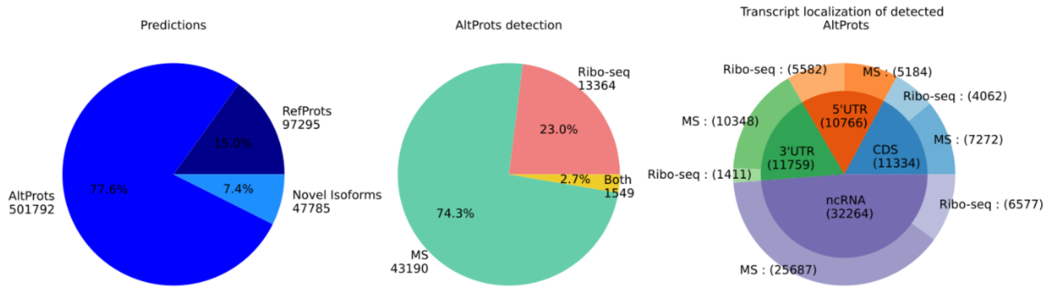

*** OpenProt v2.0 general statistics in ***Rattus norvegicus*** ***

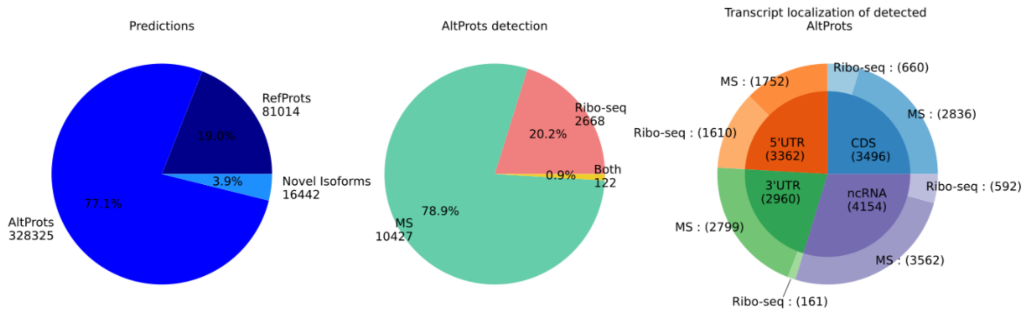

*** OpenProt v2.0 general statistics in ***Bos taurus*** ***

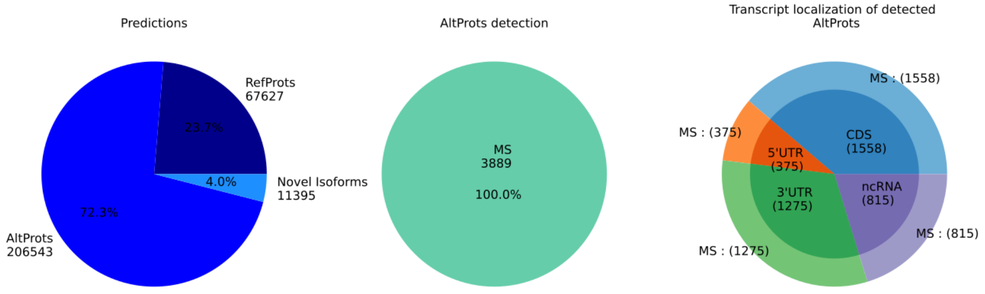

Please note that no ribosome profiling data was available for *Bos taurus*.

*** OpenProt v2.0 general statistics in ***Ovis aries*** ***

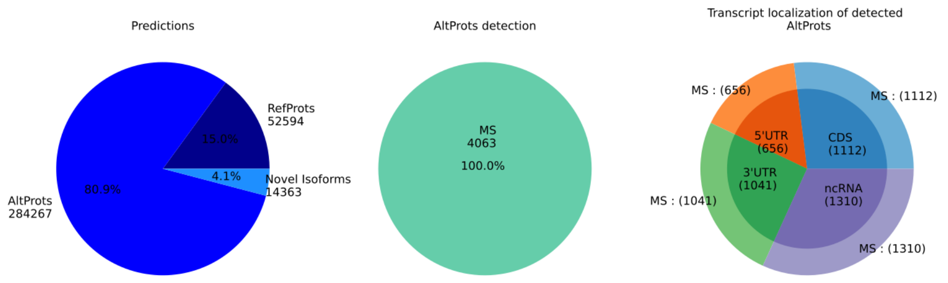

Please note that no ribosome profiling data was available for *Ovis aries*.

*** OpenProt v2.0 general statistics in ***Xenopus tropicalis*** ***

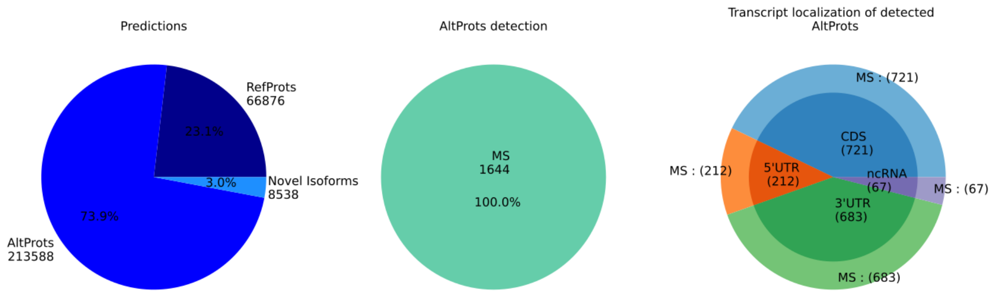

Please note that no ribosome profiling data was available for ***Xenopus tropicalis***.

*** OpenProt v2.0 general statistics in ***Danio rerio*** ***

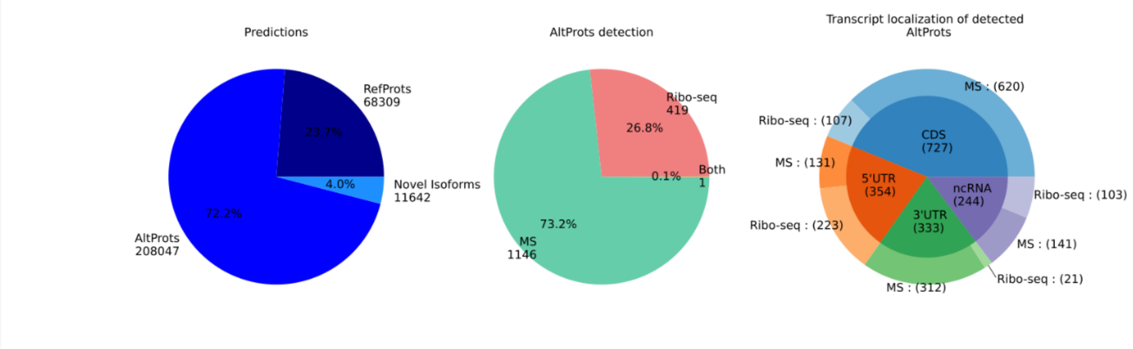

*** OpenProt v2.0 general statistics in ***Drosophila melanogaster*** ***

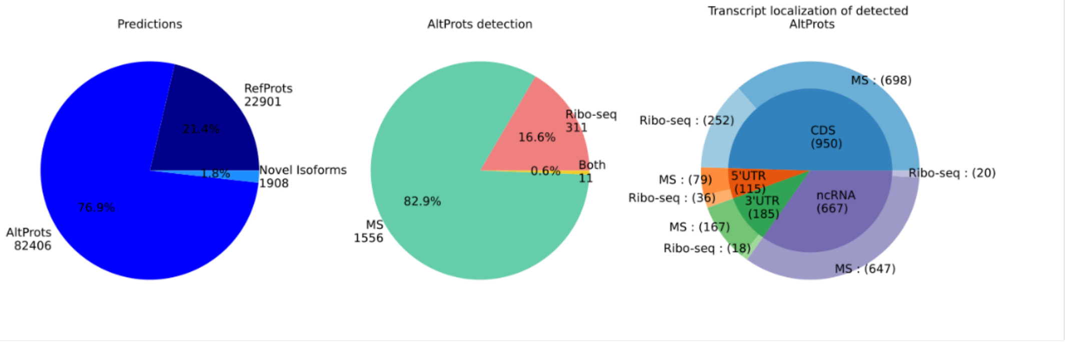

*** OpenProt v2.0 general statistics in ***Caenorhabditis elegans*** ***

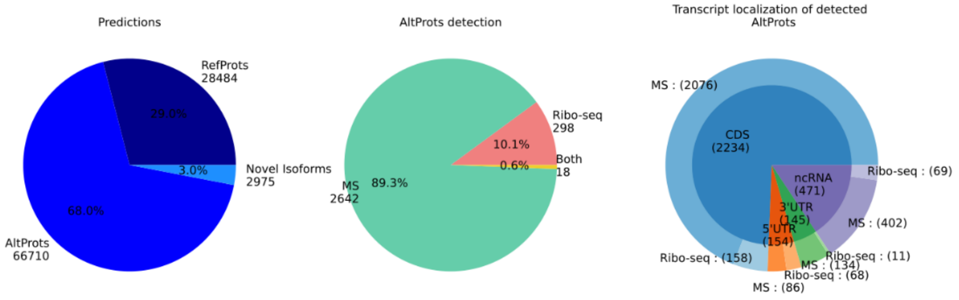

*** OpenProt v2.0 general statistics in ***Saccharomyces cerevisiae S288c*** ***

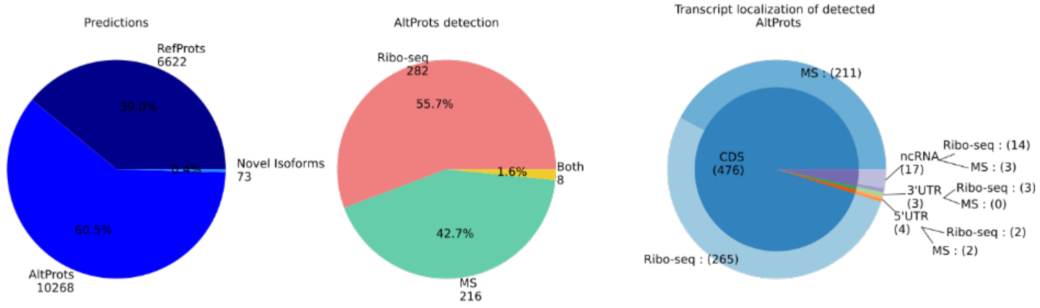

*** OpenProt v2.0 general statistics in ***Arabidopsis Thaliana*** ***

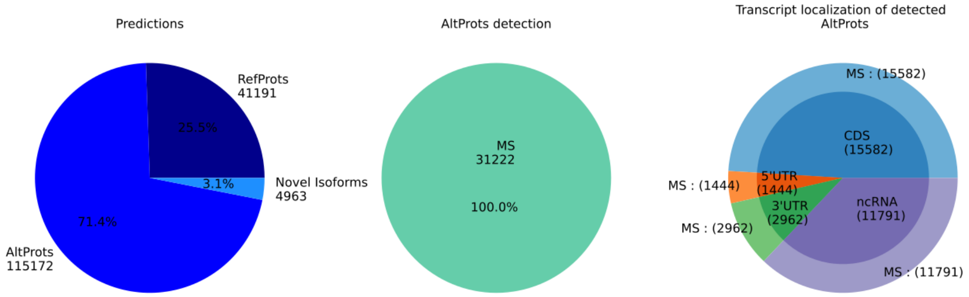

Please note that no ribosome profiling data was available for ***Arabidopsis Thaliana***.

Supplementary S3: protein structure and disorder predictions

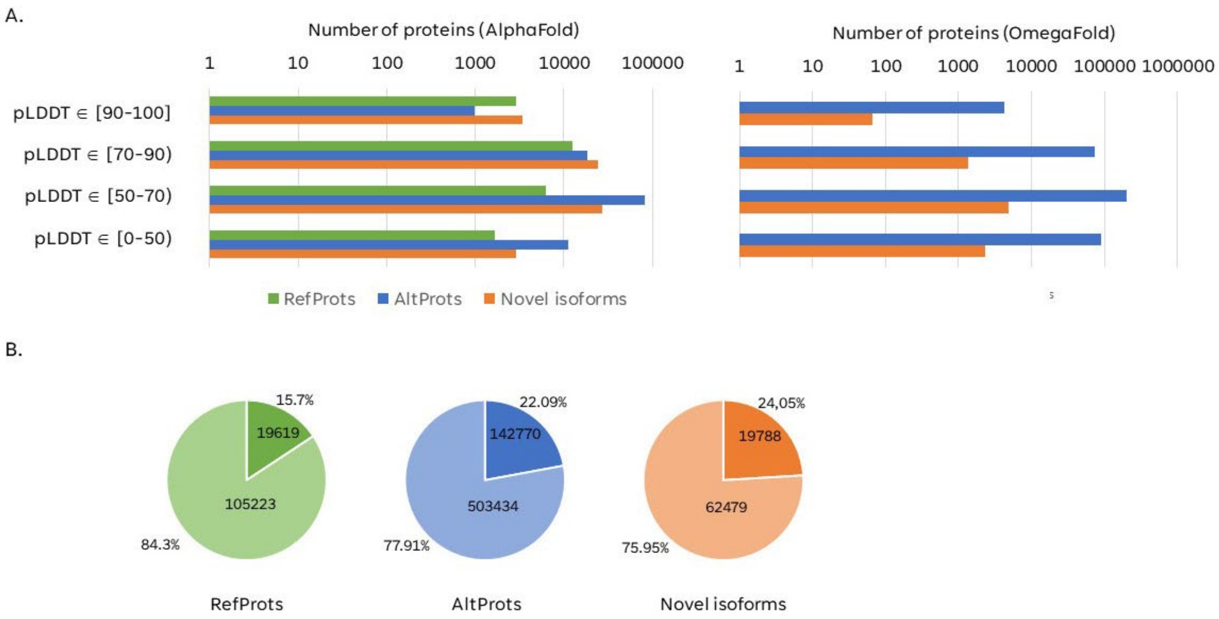

(**A**) A significant number of human AltProts and novel isoforms display a high (90 > pLDDT > 70) to very high (pLDDT >90) confidence score. Proteins with more than 30 or less than 30 multiple sequence alignments were analyzed with AlphaFold or OmegaFold, respectively. For RefProts, pLDDT scores were retrieved from the AlphaFold database. (**B**) Charts represent the fraction of proteins with at least one IDR (plain colour) or with no IDR (pale colour).

Supplementary S4

List of mass spectrometry and ribosome profiling studies incorporated in OpenProt v2.0

List of the 101 **mass spectrometry** datasets incorporated in OpenProt v2.0 with the associated species and citation (PMID accession).

Species		Study ID	Data source	PMID
Arabidopsis	thaliana	PXD013868	https://www.ebi.ac.uk/pride/archive/projects/PXD013868	32188942
Bos	taurus	PXD001741	https://www.ebi.ac.uk/pride/archive/projects/PXD001741	25818294
Caenorhabditis	elegans	PXD015644	https://www.ebi.ac.uk/pride/archive/projects/PXD015644	31754102
Danio	rerio	PXD003455	https://www.ebi.ac.uk/pride/archive/projects/PXD003455	27696471
Danio	rerio	PXD004876	https://www.ebi.ac.uk/pride/archive/projects/PXD004876	27898262
Danio	rerio	PXD011929	https://www.ebi.ac.uk/pride/archive/projects/PXD011929	32284610
Danio	rerio	PXD006098	https://www.ebi.ac.uk/pride/archive/projects/PXD006098	28381614
Drosophila	melanogaster	PXD003944	https://www.ebi.ac.uk/pride/archive/projects/PXD003944	27956707
Drosophila	melanogaster	PXD001455	https://www.ebi.ac.uk/pride/archive/projects/PXD001455	25403936
Homo	Sapiens	PXD002815	https://www.ebi.ac.uk/pride/archive/projects/PXD002815	26496610
Homo	sapiens	PXD002516	https://www.ebi.ac.uk/pride/archive/projects/PXD002516	26892330
Homo	sapiens	PXD004710	https://www.ebi.ac.uk/pride/archive/projects/PXD004710	27801565
Homo	sapiens	PXD004242	https://www.ebi.ac.uk/pride/archive/projects/PXD004242	28007936
Homo	sapiens	TCGA_COCA	https://cptc-xfer.uis.georgetown.edu/publicData/Phase_II_Data/TCGA_Colorectal_Cancer_S_022/	25043054
Homo	sapiens	PXD003965	https://www.ebi.ac.uk/pride/archive/projects/PXD003965	28208246
Homo	sapiens	PXD002214	https://www.ebi.ac.uk/pride/archive/projects/PXD	26371159

			002214	
Homo	sapiens	CCLE_2020	https://portals.broadinstitute.org/ccle/about	31978347
Homo	sapiens	PXD004778	https://www.ebi.ac.uk/pride/archive/projects/PXD004778	28071820
Homo	sapiens	PXD004900	https://www.ebi.ac.uk/pride/archive/projects/PXD004900	27806443
Homo	sapiens	PXD004764	https://www.ebi.ac.uk/pride/archive/projects/PXD004764	28071820
Homo	sapiens	TCGA_BRCA	https://cptac-data-portal.georgetown.edu/cptac/s/S030	27251275
Homo	sapiens	TCGA_OVCA	https://cptac-data-portal.georgetown.edu/cptac/s/S026	27372738
Homo	sapiens	PXD000788	https://www.ebi.ac.uk/pride/archive/projects/PXD000788	24797263
Homo	sapiens	BioPlex_1	https://bioplex.hms.harvard.edu/download.php	26186194
Homo	sapiens	PXD003708	https://www.ebi.ac.uk/pride/archive/projects/PXD003708	27987026
Homo	sapiens	PXD003902	https://www.ebi.ac.uk/pride/archive/projects/PXD003902	28007913
Homo	sapiens	PXD002854	https://www.ebi.ac.uk/pride/archive/projects/PXD002854	27135364
Homo	sapiens	PXD005083	https://www.ebi.ac.uk/pride/archive/projects/PXD005083	28196878
Homo	sapiens	PXD003947	https://www.ebi.ac.uk/pride/archive/projects/PXD003947	27444420
Homo	sapiens	PXD001889	https://www.ebi.ac.uk/pride/archive/projects/PXD001889	28248240
Homo	sapiens	PXD004541	https://www.ebi.ac.uk/pride/archive/projects/PXD004541	27827301
Homo	sapiens	PXD013647	https://www.ebi.ac.uk/pride/archive/projects/PXD013647	31513346
Homo	sapiens	PXD009208	https://www.ebi.ac.uk/pride/archive/projects/PXD009208	30157440
Homo	sapiens	PXD003940	https://www.ebi.ac.uk/pride/archive/projects/PXD003940	27976581
Homo	sapiens	PXD004625	https://www.ebi.ac.uk/pride/archive/projects/PXD004625	27879288
Homo	sapiens	PXD004437	https://www.ebi.ac.uk/pride/archive/projects/PXD004437	27890679

Homo	sapiens	PXD009668	https://www.ebi.ac.uk/pride/archive/projects/PXD009668	30607034
Homo	sapiens	PXD004624	https://www.ebi.ac.uk/pride/archive/projects/PXD004624	27879288
Homo	sapiens	PXD004875	https://www.ebi.ac.uk/pride/archive/projects/PXD004875	27779380
Homo	sapiens	PXD004788	https://www.ebi.ac.uk/pride/archive/projects/PXD004788	28071820
Homo	sapiens	BioPlex_2	https://bioplex.hms.harvard.edu/download.php	28514442
Homo	sapiens	PXD004181	https://www.ebi.ac.uk/pride/archive/projects/PXD004181	28064214
Homo	sapiens	PXD002612	https://www.ebi.ac.uk/pride/archive/projects/PXD002612	27799870
Homo	sapiens	PXD004816	https://www.ebi.ac.uk/pride/archive/projects/PXD004816	28123004
Homo	sapiens	PXD003963	https://www.ebi.ac.uk/pride/archive/projects/PXD003963	28208246
Homo	sapiens	PXD013338	https://www.ebi.ac.uk/pride/archive/projects/PXD013338	31213602
Homo	sapiens	PXD003967	https://www.ebi.ac.uk/pride/archive/projects/PXD003967	28208246
Homo	sapiens	PXD003811	https://www.ebi.ac.uk/pride/archive/projects/PXD003811	28062795
Homo	sapiens	PXD003531	https://www.ebi.ac.uk/pride/archive/projects/PXD003531	28355574
Homo	sapiens	PXD005210	https://www.ebi.ac.uk/pride/archive/projects/PXD005210	27794609
Homo	sapiens	PXD001994	https://www.ebi.ac.uk/pride/archive/projects/PXD001994	26832662
Homo	sapiens	PXD005123	https://www.ebi.ac.uk/pride/archive/projects/PXD005123	27892468
Homo	sapiens	PXD003971	https://www.ebi.ac.uk/pride/archive/projects/PXD003971	28208246
Homo	sapiens	PXD005276	https://www.ebi.ac.uk/pride/archive/projects/PXD005276	28237943
Homo	sapiens	PXD004859	https://www.ebi.ac.uk/pride/archive/projects/PXD004859	27976366
Homo	sapiens	PXD004796	https://www.ebi.ac.uk/pride/archive/projects/PXD004796	28071820
Homo	sapiens	PXD016433	https://www.ebi.ac.uk/pride/archive/projects/PXD	33453410

			016433	
Homo	sapiens	PXD003966	https://www.ebi.ac.uk/pride/archive/projects/PXD003966	28208246
Homo	sapiens	PXD005739	https://www.ebi.ac.uk/pride/archive/projects/PXD005739	28648085
Homo	sapiens	PXD010603	https://www.ebi.ac.uk/pride/archive/projects/PXD010603	30735395
Homo	sapiens	PXD004626	https://www.ebi.ac.uk/pride/archive/projects/PXD004626	27879288
Homo	sapiens	PXD017417	https://www.ebi.ac.uk/pride/archive/projects/PXD017417	32966781
Homo	sapiens	PXD010138	https://www.ebi.ac.uk/pride/archive/projects/PXD010138	31207390
Homo	sapiens	PXD000419	https://www.ebi.ac.uk/pride/archive/projects/PXD000419	24136357
Homo	sapiens	PXD015850	https://www.ebi.ac.uk/pride/archive/projects/PXD015850	31614365
Mus	musculus	PXD000288	https://www.ebi.ac.uk/pride/archive/projects/PXD000288	25616865
Mus	musculus	PXD001597	https://www.ebi.ac.uk/pride/archive/projects/PXD001597	26227174
Mus	musculus	PXD003155	https://www.ebi.ac.uk/pride/archive/projects/PXD003155	26850065
Mus	musculus	PXD002156	https://www.ebi.ac.uk/pride/archive/projects/PXD002156	26080680
Mus	musculus	PXD001250	https://www.ebi.ac.uk/pride/archive/projects/PXD001250	26523646
Mus	musculus	PXD004072	https://www.ebi.ac.uk/pride/archive/projects/PXD004072	27188442
Mus	musculus	PXD002152	https://www.ebi.ac.uk/pride/archive/projects/PXD002152	27302655
Mus	musculus	PXD001792	https://www.ebi.ac.uk/pride/archive/projects/PXD001792	26280412
Mus	musculus	PXD005312	https://www.ebi.ac.uk/pride/archive/projects/PXD005312	28066266
Mus	musculus	PXD003860	https://www.ebi.ac.uk/pride/archive/projects/PXD003860	27432266
Mus	musculus	PXD004612	https://www.ebi.ac.uk/pride/archive/projects/PXD004612	28071813
Mus	musculus	PXD017284	https://www.ebi.ac.uk/pride/archive/projects/PXD	33257503

			017284	
Mus	musculus	PXD001293	https://www.ebi.ac.uk/pride/archive/projects/PXD001293	25504905
Mus	musculus	PXD000666	https://www.ebi.ac.uk/pride/archive/projects/PXD000666	25193168
Mus	musculus	PXD005635	https://www.ebi.ac.uk/pride/archive/projects/PXD005635	28345880
Mus	musculus	PXD011890	https://www.ebi.ac.uk/pride/archive/projects/PXD011890	30901149
Mus	musculus	PXD003442	https://www.ebi.ac.uk/pride/archive/projects/PXD003442	26900923
Mus	musculus	PXD003555	https://www.ebi.ac.uk/pride/archive/projects/PXD003555	27490109
Mus	musculus	PXD000747	https://www.ebi.ac.uk/pride/archive/projects/PXD000747	25205226
Mus	musculus	PXD004087	https://www.ebi.ac.uk/pride/archive/projects/PXD004087	27629805
Mus	musculus	PXD011334	https://www.ebi.ac.uk/pride/archive/projects/PXD011334	33216553
Mus	musculus	PXD000501	https://www.ebi.ac.uk/pride/archive/projects/PXD000501	24753479
Mus	musculus	PXD001514	https://www.ebi.ac.uk/pride/archive/projects/PXD001514	26139848
Mus	musculus	PXD001404	https://www.ebi.ac.uk/pride/archive/projects/PXD001404	25338131
Ovis	aries	PXD014050	https://www.ebi.ac.uk/pride/archive/projects/PXD014050	31882954
Ovis	aries	PXD013822	https://www.ebi.ac.uk/pride/archive/projects/PXD013822	31136077
Ovis	aries	PXD004989	https://www.ebi.ac.uk/pride/archive/projects/PXD004989	28615994
Ovis	aries	PXD004556	https://www.ebi.ac.uk/pride/archive/projects/PXD004556	27784645
Rattus	norvegicus	PXD004247	https://www.ebi.ac.uk/pride/archive/projects/PXD004247	27250205
Rattus	norvegicus	PXD001984	https://www.ebi.ac.uk/pride/archive/projects/PXD001984	26330543
Rattus	norvegicus	PXD001986	https://www.ebi.ac.uk/pride/archive/projects/PXD001986	26330543
Rattus	norvegicus	PXD004889	https://www.ebi.ac.uk/pride/archive/projects/PXD	27764671

			004889	
Rattus	norvegicus	PXD016447	https://www.ebi.ac.uk/pride/archive/projects/PXD016447	33453410
Rattus	norvegicus	PXD003520	https://www.ebi.ac.uk/pride/archive/projects/PXD003520	27358910
Rattus	norvegicus	PXD003375	https://www.ebi.ac.uk/pride/archive/projects/PXD003375	27250205
Xenopus	tropicalis	PXD009577	https://www.ebi.ac.uk/pride/archive/projects/PXD009577	31490923

List of the 136 **ribosome profiling** datasets incorporated in OpenProt v2.0 along the associated species, data source and citation (PMID accession).

Species	Study ID	Data source	PMID
CE	GSE52910	https://www.ncbi.nlm.nih.gov/geo/query/acc.cgi?acc=GSE52910	24440504
CE	GSE62859	https://www.ncbi.nlm.nih.gov/geo/query/acc.cgi?acc=GSE62859	25378320
CE	SRP014427	https://www.ncbi.nlm.nih.gov/sra/?term=SRP014427	22855835
DM	GSE49197	https://www.ncbi.nlm.nih.gov/geo/query/acc.cgi?acc=GSE49197	24302569
DM	GSE52799	https://www.ncbi.nlm.nih.gov/geo/query/acc.cgi?acc=GSE52799	24882012
DM	GSE60384	https://www.ncbi.nlm.nih.gov/geo/query/acc.cgi?acc=GSE60384	25144939
DM	GSE106697	https://www.ncbi.nlm.nih.gov/geo/query/acc.cgi?acc=GSE106697	29548011
DR	GSE34743	https://www.ncbi.nlm.nih.gov/geo/query/acc.cgi?acc=GSE34743	22422859
DR	GSE46512	https://www.ncbi.nlm.nih.gov/geo/query/acc.cgi?acc=GSE46512	23698349
DR	GSE47558	https://www.ncbi.nlm.nih.gov/geo/query/acc.cgi?acc=GSE47558	24056933
DR	GSE52809	https://www.ncbi.nlm.nih.gov/geo/query/acc.cgi?acc=GSE52809	24476825
DR	GSE53693	https://www.ncbi.nlm.nih.gov/geo/query/acc.cgi?acc=GSE53693	24705786
HS	GSE102113	https://www.ncbi.nlm.nih.gov/geo/query/acc.cgi?acc=GSE102113	30449621
HS	GSE103719	https://www.ncbi.nlm.nih.gov/geo/query/acc.cgi?acc=GSE103719	30257221
HS	GSE105082	https://www.ncbi.nlm.nih.gov/geo/query/acc.cgi?acc=GSE105082	30591072
HS	GSE105172	https://www.ncbi.nlm.nih.gov/geo/query/acc.cgi?acc=GSE105172	31160600
HS	GSE110323	https://www.ncbi.nlm.nih.gov/geo/query/acc.cgi?acc=GSE110323	30867593
HS	GSE111866	https://www.ncbi.nlm.nih.gov/geo/query/acc.cgi?acc=GSE111866	30102689
HS	GSE112276	https://www.ncbi.nlm.nih.gov/geo/query/acc.cgi?acc=GSE112276	31031084
HS	GSE112353	https://www.ncbi.nlm.nih.gov/geo/query/acc.cgi?acc=GSE112353	30355487
HS	GSE115146	https://www.ncbi.nlm.nih.gov/geo/query/acc.cgi?acc=GSE115146	30297778
HS	GSE118050	https://www.ncbi.nlm.nih.gov/geo/query/acc.cgi?acc=GSE118050	30260431
HS	GSE122461	https://www.ncbi.nlm.nih.gov/geo/query/acc.cgi?acc=GSE122461	30640896
HS	GSE123564	https://www.ncbi.nlm.nih.gov/geo/query/acc.cgi?acc=GSE123564	30707697
HS	GSE125218	https://www.ncbi.nlm.nih.gov/geo/query/acc.cgi?acc=GSE125218	31819274
HS	GSE129869	https://www.ncbi.nlm.nih.gov/geo/query/acc.cgi?acc=GSE129869	31167946
HS	GSE130781	https://www.ncbi.nlm.nih.gov/geo/query/acc.cgi?acc=GSE130781	31944153
HS	GSE131112	https://www.ncbi.nlm.nih.gov/geo/query/acc.cgi?acc=GSE131112	31284728
HS	GSE131809	https://www.ncbi.nlm.nih.gov/geo/query/acc.cgi?acc=GSE131809	31959994
HS	GSE132725	https://www.ncbi.nlm.nih.gov/geo/query/acc.cgi?acc=GSE132725	31340047
HS	GSE133111	https://www.ncbi.nlm.nih.gov/geo/query/acc.cgi?acc=GSE133111	
HS	GSE134752	https://www.ncbi.nlm.nih.gov/geo/query/acc.cgi?acc=GSE134752	32348767
HS	GSE37744	https://www.ncbi.nlm.nih.gov/geo/query/acc.cgi?acc=GSE37744	22836135
HS	GSE39561	https://www.ncbi.nlm.nih.gov/geo/query/acc.cgi?acc=GSE39561	22879431
HS	GSE41605	https://www.ncbi.nlm.nih.gov/geo/query/acc.cgi?acc=GSE41605	23180859
HS	GSE45833	https://www.ncbi.nlm.nih.gov/geo/query/acc.cgi?acc=GSE45833	23594524
HS	GSE48785	https://www.ncbi.nlm.nih.gov/geo/query/acc.cgi?acc=GSE48785	24171104
HS	GSE48933	https://www.ncbi.nlm.nih.gov/geo/query/acc.cgi?acc=GSE48933	24301020
HS	GSE49339	https://www.ncbi.nlm.nih.gov/geo/query/acc.cgi?acc=GSE49339	23453015
HS	GSE51424	https://www.ncbi.nlm.nih.gov/geo/query/acc.cgi?acc=GSE51424	25122893
HS	GSE51584	https://www.ncbi.nlm.nih.gov/geo/query/acc.cgi?acc=GSE51584	25070500
HS	GSE52976	https://www.ncbi.nlm.nih.gov/geo/query/acc.cgi?acc=GSE52976	25366541
HS	GSE55195	https://www.ncbi.nlm.nih.gov/geo/query/acc.cgi?acc=GSE55195	25621764
HS	GSE56887	https://www.ncbi.nlm.nih.gov/geo/query/acc.cgi?acc=GSE56887	25079319
HS	GSE56924	https://www.ncbi.nlm.nih.gov/geo/query/acc.cgi?acc=GSE56924	26338483
HS	GSE58207	https://www.ncbi.nlm.nih.gov/geo/query/acc.cgi?acc=GSE58207	25510491
HS	GSE59821	https://www.ncbi.nlm.nih.gov/geo/query/acc.cgi?acc=GSE59821	26878238
HS	GSE60040	https://www.ncbi.nlm.nih.gov/geo/query/acc.cgi?acc=GSE60040	25989971
HS	GSE60095	https://www.ncbi.nlm.nih.gov/geo/query/acc.cgi?acc=GSE60095	25159147

HS	GSE61375	https://www.ncbi.nlm.nih.gov/geo/query/acc.cgi?acc=GSE61375	25273840
HS	GSE62247	https://www.ncbi.nlm.nih.gov/geo/query/acc.cgi?acc=GSE62247	26399832
HS	GSE63570	https://www.ncbi.nlm.nih.gov/geo/query/acc.cgi?acc=GSE63570	25896322
HS	GSE64962	https://www.ncbi.nlm.nih.gov/geo/query/acc.cgi?acc=GSE64962	26729373
HS	GSE65778	https://www.ncbi.nlm.nih.gov/geo/query/acc.cgi?acc=GSE65778	25719440
HS	GSE65885	https://www.ncbi.nlm.nih.gov/geo/query/acc.cgi?acc=GSE65885	26687005
HS	GSE65912	https://www.ncbi.nlm.nih.gov/geo/query/acc.cgi?acc=GSE65912	26297486
HS	GSE66927	https://www.ncbi.nlm.nih.gov/geo/query/acc.cgi?acc=GSE66927	26538417
HS	GSE67902	https://www.ncbi.nlm.nih.gov/geo/query/acc.cgi?acc=GSE67902	26305499
HS	GSE68008	https://www.ncbi.nlm.nih.gov/geo/query/acc.cgi?acc=GSE68008	26164698
HS	GSE69906	https://www.ncbi.nlm.nih.gov/geo/query/acc.cgi?acc=GSE69906	26599541
HS	GSE70211	https://www.ncbi.nlm.nih.gov/geo/query/acc.cgi?acc=GSE70211	27309803
HS	GSE70802	https://www.ncbi.nlm.nih.gov/geo/query/acc.cgi?acc=GSE70802	27058758
HS	GSE73136	https://www.ncbi.nlm.nih.gov/geo/query/acc.cgi?acc=GSE73136	26657557
HS	GSE73565	https://www.ncbi.nlm.nih.gov/geo/query/acc.cgi?acc=GSE73565	26898226
HS	GSE75290	https://www.ncbi.nlm.nih.gov/geo/query/acc.cgi?acc=GSE75290	27232982
HS	GSE79664	https://www.ncbi.nlm.nih.gov/geo/query/acc.cgi?acc=GSE79664	27153541
HS	GSE85864	https://www.ncbi.nlm.nih.gov/geo/query/acc.cgi?acc=GSE85864	27681415
HS	GSE86214	https://www.ncbi.nlm.nih.gov/geo/query/acc.cgi?acc=GSE86214	28106072
HS	GSE93133	https://www.ncbi.nlm.nih.gov/geo/query/acc.cgi?acc=GSE93133	28108655
HS	GSE94460	https://www.ncbi.nlm.nih.gov/geo/query/acc.cgi?acc=GSE94460	29170441
HS	GSE97140	https://www.ncbi.nlm.nih.gov/geo/query/acc.cgi?acc=GSE97140	28520920
HS	GSE97384	https://www.ncbi.nlm.nih.gov/geo/query/acc.cgi?acc=GSE97384	28494858
HS	SRA049309	https://www.ncbi.nlm.nih.gov/sra/?term=SRA049309	22045228
HS	SRA056377	https://www.ncbi.nlm.nih.gov/sra/?term=SRA056377	22927429
HS	SRA061778	https://www.ncbi.nlm.nih.gov/sra/?term=SRA061778	23290916
HS	GSE63591	https://www.ncbi.nlm.nih.gov/sra/?term=SRA099816	26046440
HS	SRA099816	https://www.ncbi.nlm.nih.gov/sra/?term=SRA099816	24120665
HS	GSE74279	https://www.ncbi.nlm.nih.gov/geo/query/acc.cgi?acc=GSE74279	26893308
HS	GSE56148	https://www.ncbi.nlm.nih.gov/geo/query/acc.cgi?acc=GSE56148	25108525
HS	GSE131111	https://www.ncbi.nlm.nih.gov/geo/query/acc.cgi?acc=GSE131111	31284728
HS	GSE124558	https://www.ncbi.nlm.nih.gov/geo/query/acc.cgi?acc=GSE124558	31819270
HS	GSE141013	https://www.ncbi.nlm.nih.gov/geo/query/acc.cgi?acc=GSE141013	32488114
HS	GSE136664	https://www.ncbi.nlm.nih.gov/geo/query/acc.cgi?acc=GSE136664	32064749
HS	GSE121391	https://www.ncbi.nlm.nih.gov/geo/query/acc.cgi?acc=GSE121391	30819820
HS	GSE66810	https://www.ncbi.nlm.nih.gov/geo/query/acc.cgi?acc=GSE66810	26147685
MM	GSE103667	https://www.ncbi.nlm.nih.gov/geo/query/acc.cgi?acc=GSE103667	29576526
MM	GSE105147	https://www.ncbi.nlm.nih.gov/geo/query/acc.cgi?acc=GSE105147	30643286
MM	GSE106529	https://www.ncbi.nlm.nih.gov/geo/query/acc.cgi?acc=GSE106529	31186416
MM	GSE115106	https://www.ncbi.nlm.nih.gov/geo/query/acc.cgi?acc=GSE115106	30728504
MM	GSE115110	https://www.ncbi.nlm.nih.gov/geo/query/acc.cgi?acc=GSE115110	31296853
MM	GSE119365	https://www.ncbi.nlm.nih.gov/geo/query/acc.cgi?acc=GSE119365	31033440
MM	GSE124280	https://www.ncbi.nlm.nih.gov/geo/query/acc.cgi?acc=GSE124280	31924774
MM	GSE129816	https://www.ncbi.nlm.nih.gov/geo/query/acc.cgi?acc=GSE129816	31483294
MM	GSE129818	https://www.ncbi.nlm.nih.gov/geo/query/acc.cgi?acc=GSE129818	31483294
MM	GSE130898	https://www.ncbi.nlm.nih.gov/geo/query/acc.cgi?acc=GSE130898	31371437
MM	GSE141599	https://www.ncbi.nlm.nih.gov/geo/query/acc.cgi?acc=GSE141599	31999954
MM	GSE142802	https://www.ncbi.nlm.nih.gov/geo/query/acc.cgi?acc=GSE142802	32123389
MM	GSE22004	https://www.ncbi.nlm.nih.gov/geo/query/acc.cgi?acc=GSE22004	20703300
MM	GSE30839	https://www.ncbi.nlm.nih.gov/geo/query/acc.cgi?acc=GSE30839	22056041
MM	GSE36892	https://www.ncbi.nlm.nih.gov/geo/query/acc.cgi?acc=GSE36892	22552098
MM	GSE41785	https://www.ncbi.nlm.nih.gov/geo/query/acc.cgi?acc=GSE41785	23766421
MM	GSE46038	https://www.ncbi.nlm.nih.gov/geo/query/acc.cgi?acc=GSE46038	23696641

MM	GSE50983	https://www.ncbi.nlm.nih.gov/geo/query/acc.cgi?acc=GSE50983	25063675
MM	GSE53743	https://www.ncbi.nlm.nih.gov/geo/query/acc.cgi?acc=GSE53743	25215492
MM	GSE58423	https://www.ncbi.nlm.nih.gov/geo/query/acc.cgi?acc=GSE58423	25380226
MM	GSE59793	https://www.ncbi.nlm.nih.gov/geo/query/acc.cgi?acc=GSE59793	25745177
MM	GSE60426	https://www.ncbi.nlm.nih.gov/geo/query/acc.cgi?acc=GSE60426	25263593
MM	GSE67305	https://www.ncbi.nlm.nih.gov/geo/query/acc.cgi?acc=GSE67305	26486724
MM	GSE69800	https://www.ncbi.nlm.nih.gov/geo/query/acc.cgi?acc=GSE69800	27161320
MM	GSE72066	https://www.ncbi.nlm.nih.gov/geo/query/acc.cgi?acc=GSE72066	27306184
MM	GSE72851	https://www.ncbi.nlm.nih.gov/geo/query/acc.cgi?acc=GSE72851	28225755
MM	GSE73369	https://www.ncbi.nlm.nih.gov/geo/query/acc.cgi?acc=GSE73369	26443847
MM	GSE74139	https://www.ncbi.nlm.nih.gov/geo/query/acc.cgi?acc=GSE74139	26638175
MM	GSE74683	https://www.ncbi.nlm.nih.gov/geo/query/acc.cgi?acc=GSE74683	26651292
MM	GSE83332	https://www.ncbi.nlm.nih.gov/geo/query/acc.cgi?acc=GSE83332	28077873
MM	GSE83823	https://www.ncbi.nlm.nih.gov/geo/query/acc.cgi?acc=GSE83823	27899360
MM	GSE84112	https://www.ncbi.nlm.nih.gov/geo/query/acc.cgi?acc=GSE84112	27956496
MM	GSE89108	https://www.ncbi.nlm.nih.gov/geo/query/acc.cgi?acc=GSE89108	28720757
MM	PRJEB7207	http://www.ebi.ac.uk/ena/data/view/PRJEB7207	25873627
MM	SRA160745	https://www.ncbi.nlm.nih.gov/sra/?term=SRA160745	25486063
MM	GSE62134	https://www.ncbi.nlm.nih.gov/geo/query/acc.cgi?acc=GSE62134	25706746
MM	GSE72064	https://www.ncbi.nlm.nih.gov/geo/query/acc.cgi?acc=GSE72064	26430118
MM	GSE65786	https://www.ncbi.nlm.nih.gov/geo/query/acc.cgi?acc=GSE65786	32015435
MM	GSE116083	https://www.ncbi.nlm.nih.gov/geo/query/acc.cgi?acc=GSE116083	31366581
MM	GSE116983	https://www.ncbi.nlm.nih.gov/geo/query/acc.cgi?acc=GSE116983	30420357
RN	GSE110426	https://www.ncbi.nlm.nih.gov/geo/query/acc.cgi?acc=GSE110426	29878763
RN	GSE66715	https://www.ncbi.nlm.nih.gov/geo/query/acc.cgi?acc=GSE66715	27135913
RN	GSE60752	https://www.ncbi.nlm.nih.gov/geo/query/acc.cgi?acc=GSE60752	25943107
RN	ERP007231	https://www.ncbi.nlm.nih.gov/sra/?term=ERP007231	26007203
SC	GSE34082	https://www.ncbi.nlm.nih.gov/geo/query/acc.cgi?acc=GSE34082	22194413
SC	GSE45366	https://www.ncbi.nlm.nih.gov/geo/query/acc.cgi?acc=GSE45366	23935536
SC	GSE61012	https://www.ncbi.nlm.nih.gov/geo/query/acc.cgi?acc=GSE61012	25378630
SC	GSE63789	https://www.ncbi.nlm.nih.gov/geo/query/acc.cgi?acc=GSE63789	25538139
SC	GSE67387	https://www.ncbi.nlm.nih.gov/geo/query/acc.cgi?acc=GSE67387	26052047
SC	GSE75322	https://www.ncbi.nlm.nih.gov/geo/query/acc.cgi?acc=GSE75322	26887592
SC	GSE82220	https://www.ncbi.nlm.nih.gov/geo/query/acc.cgi?acc=GSE82220	27638886
